# Supplementary material for: Size control of the inner ear via hydraulic feedback
Source: eLife. 2019 Oct 1;8:e39596. doi: 10.7554/eLife.39596 (PMC6773445; doi:10.7554/eLife.39596)
Supplement: Transparent reporting form [file elife-39596-transrepform.docx]

***eLife’s* transparent reporting form**

We encourage authors to provide detailed information *within their submission* to facilitate the interpretation and replication of experiments. Authors can upload supporting documentation to indicate the use of appropriate reporting guidelines for health-related research (see [EQUATOR Network](http://www.equator-network.org/%20)), life science research (see the [BioSharing Information Resource](https://biosharing.org/)), or the [ARRIVE guidelines](http://www.plosbiology.org/article/info:doi/10.1371/journal.pbio.1000412) for reporting work involving animal research. Where applicable, authors should refer to any relevant reporting standards documents in this form.

If you have any questions, please consult our Journal Policies and/or contact us: [editorial@elifesciences.org](mailto:editorial@elifesciences.org).

**Sample-size estimation**

- You should state whether an appropriate sample size was computed when the study was being designed
- You should state the statistical method of sample size computation and any required assumptions
- If no explicit power analysis was used, you should describe how you decided what sample (replicate) size (number) to use

Please outline where this information can be found within the submission (e.g., sections or figure legends), or explain why this information doesn’t apply to your submission:

Since this work studied a novel phenomenon and was therefore highly driven by observations, we do not explicitly define the sample size at the start of the study. We also note that the experimental approach and image analyses were time-consuming and labor intensive, which limits the sample size in some tests. However, the standard statistical hypothesis test was performed to ensure the sample size is sufficiently large to support significant results.

The morphodynamic quantifications in Figure 1 came from 10 different embryos for each time-point, 30 time-points were analyzed, to include 300 lumen measurements and more than 200,000 segmented cells. The experiments of figure 1 are not hypothesis based, so calculating sample size is not appropriate. The goal of these experiments was to quantify the phenomenon of otic vesicle growth with the best possible precision and accuracy and towards that goal we oversampled. However, because this was not hypothesis based, it was not possible to calculate sample size for unknown or potentially rare phenomenon.

Figure 2 presents the quantification of an unexpected phenomenon of deformations of mitotic cell shapes and neighboring tissue, which together indicated the presence of pressure-driven strain. We observed 54 mitotic cells while imaging 60 different ears. The data is helping to dismiss the null hypothesis that fluid accumulation might occur without the building up of pressure. Figure 2G, we used 5-10 embryos per timepoint and we identified a total of 54 mitotic events across all the timepoints. There were 0-2 mitotic events spotted per embryo. The data is significantly different from 22hpf (∗p<1.0e-4), and 27 hpf (∗∗p<1.0e-5), as determined using student t-test, unpaired.

Figure 3 directly confirms the build-up of luminal pressure. Figure 3C reports the use of a pressure sensor directly measured an increase in pressure, from undetectable at 24 hpf to >200 Pa at 48 hpf, which is much greater than the sensors precision of +/- 5 Pa. 5 to 9 measurements were performed at each time-point, which is sufficient to reject the null-hypothesis that there is no build-up of pressure in a student t-test(∗p<5.0e-2,).

Figure 3E-G report another indirect observation of the build-up of hydrostatic pressure by measuring the otic vesicle’s response to puncture. Each datapoint is 10 measurements and the statistical significance of differences was determined using a student t-test.

Figure 4A present the result of calculating the fluid flux during otic vesicle growth. The segmented lumen volumes generated in Figure 1 were fit to quadratic polynomials to determine the rate of volume change divided by surface area of the lumen. This plot is derived from 300 measurements that exhibited little variation.

The data points in Figure 4G-H are each derived from 10 otic vesicles. The significance of the difference between punctured and unpunctured in G and H were tested using student t-test.

In figure 4G we report that the difference in fluid flux between punctured and unpunctured as a function of time is significant at 32.5 hpf with p<1.0e-4 and at 37.5 hpf with p<1.0e-5.

In figure 4H we report that the difference in fluid flux between punctured and unpunctured as a function of volume ratio is significant at volume ratios of 0.5 and 0.8 with p<1.0e-5.

For Figure 5, each data point is derived from 5 measurements.

In figure 5A, we report that the volume differences are signiﬁcant with p<1.0e5 for both WT and WT+DMSO vs. 0.1, 0.5, and 1mM oubain treatments. Additionally, tissue thickness differences are signiﬁcant with p<1.0e-5 for both WT and WT+DMSO vs. 0.5, and 1mM oubain treatments. These differences were tested using a student t-test.

In figure 5E, we report the quantiﬁcation of lumenal volumes at 30 hpf after acidiﬁcation of buffer (pH of 6.5-8.0) at 12 hpf. Volume differences between 7.0 vs 7.5 pH treatment are signiﬁcant with p<1.0e-3. Volume differences between 7.0 vs 8.0 pH treatment are signiﬁcant with p<1.0e-5 using student t-test.

In figure 5F we report a dose-dependent decrease in lumenal volumes after the addition of niﬂumic acid, a chloride channel inhibitor at 12 hpf (blue) or 20 hpf (red). n=5 for each data point, differences between WT and niﬂumic

acid treatments of 1, 10, and 100 µM are signiﬁcant with p<1.0e-5 using a student t-test.

For Figure 6B we report normalized change in wall thickness post-puncture near the hindbrain (medial, blue), ectoderm (lateral, red), and anteroposterior regions (poles, green) and find change in thickness differences between pole and medial cells and pole and lateral cells are significant with p<1.0e-5 at 25 and 30 hpf and p<1.0e-3 at 35hpf, as determined using a student t-test. Each data point is derived from 5 measurements.

For Figure 6C we report resting wall thickness post-puncture near the hindbrain (medial, blue), ectoderm (lateral, red), and anteroposterior regions (poles, green) and find change in thickness differences between pole and medial cells and pole and lateral cells are significant with p<1.0e-5 at 27.5 and 32.5 hpf, as determined using a student t-test. Each data point is derived from 5 measurements.

**Replicates**

- You should report how often each experiment was performed
- You should include a definition of biological versus technical replication
- The data obtained should be provided and sufficient information should be provided to indicate the number of independent biological and/or technical replicates
- If you encountered any outliers, you should describe how these were handled
- Criteria for exclusion/inclusion of data should be clearly stated
- High-throughput sequence data should be uploaded before submission, with a private link for reviewers provided (these are available from both GEO and ArrayExpress)

Please outline where this information can be found within the submission (e.g., sections or figure legends), or explain why this information doesn’t apply to your submission:

The number of replicates are reported in each figure legend.

The Criteria for exclusion/inclusion of data for pressure measurement is detailed in the METHOD DETAILS session “Vesicular fluid pressure probe”.

**Statistical reporting**

- Statistical analysis methods should be described and justified
- Raw data should be presented in figures whenever informative to do so (typically when N per group is less than 10)
- For each experiment, you should identify the statistical tests used, exact values of N, definitions of center, methods of multiple test correction, and dispersion and precision measures (e.g., mean, median, SD, SEM, confidence intervals; and, for the major substantive results, a measure of effect size (e.g., Pearson's r, Cohen's d)
- Report exact p-values wherever possible alongside the summary statistics and 95% confidence intervals. These should be reported for all key questions and not only when the p-value is less than 0.05.

Please outline where this information can be found within the submission (e.g., sections or figure legends), or explain why this information doesn’t apply to your submission:

This information is present in the Figures and figure legends.

(For large datasets, or papers with a very large number of statistical tests, you may upload a single table file with tests, Ns, etc., with reference to sections in the manuscript.)

**Group allocation**

- Indicate how samples were allocated into experimental groups (in the case of clinical studies, please specify allocation to treatment method); if randomization was used, please also state if restricted randomization was applied
- Indicate if masking was used during group allocation, data collection and/or data analysis

Please outline where this information can be found within the submission (e.g., sections or figure legends), or explain why this information doesn’t apply to your submission:

Samples were either perturbed or unperturbed or different developmental stages. Whenever possible, we used the contralateral ear as an internal control. No masking was using during group allocation.

**Additional data files (“source data”)**

- We encourage you to upload relevant additional data files, such as numerical data that are represented as a graph in a figure, or as a summary table
- Where provided, these should be in the most useful format, and they can be uploaded as “Source data” files linked to a main figure or table
- Include model definition files including the full list of parameters used
- Include code used for data analysis (e.g., R, MatLab)
- Avoid stating that data files are “available upon request”

Please indicate the figures or tables for which source data files have been provided:

The complete model is presented in the Methods section.

All the raw data for the pressure measurement can be found in Figure 3–figure supplement 2.
